# European SMEs amidst the COVID-19 crisis: assessing impact and policy responses

**DOI:** 10.1007/s40812-020-00169-4

**Published:** 2020-07-11

**Authors:** Jill Juergensen, José Guimón, Rajneesh Narula

**Affiliations:** 1 Henley Business School, University of Reading, Whiteknights, Reading, RG6 6UD UK; 2 Department of Development Economics, Autonomous University of Madrid, Campus de Cantoblanco, 28049 Madrid, Spain

**Keywords:** European SMEs, Innovation, COVID-19, Economic impact, Policy responses

## Abstract

We consider how the COVID-19 pandemic has challenged European small- and medium-sized enterprises (SMEs) in the manufacturing sector, and draw suggests policy implications. The sudden onslaught of the pandemic has acted as an economic shock, and we consider how it is likely to affect different types of manufacturing SMEs. We distinguish between immediate effects, a result of the almost-simultaneous lockdowns across Europe and its major trading partners, and longer-term implications for both SMEs and the global value chains where they are inserted. In the shorter run, most SMEs have faced logistical challenges in addition to demand disruptions, although the severity has differed across firms and industries. We argue that in the longer-term, there will be different challenges and opportunities depending on the type of SME. Policy interventions will also need to be sensitive to the different types of SMEs, rather than adopting a one-size-fits-all approach. The policy mix will need to shift from its initial focus on the survival of European SMEs in the short term, towards a more structural and longer-term approach based on promoting their renewal and growth through innovation, internationalization and networking.

## Introduction

Small- and medium-sized enterprises (SMEs)^1^ are the backbone of the European economy, making up 99.8% of all enterprises and two-thirds of employment (European Commission 2019). In the case of the manufacturing sector, which forms the focus of this paper, SMEs account for 58% of employment and 42% of value added (European Commission 2017). Over the last few decades, global value chains (GVCs) have expanded amidst the growing fragmentation of economic activity and the concurrent decline of the vertically integrated firm (Kano et al. 2020). The flexibility of GVCs and their ability to reorganize themselves to optimize efficiency has proven challenging for European SMEs, in part, due to high labor costs and rigid regulations. However, the increased application of digital technologies in manufacturing, also known as Industry 4.0 (van Tulder et al. 2018), has been interpreted as a new opportunity for Europe that may enable a renaissance of local manufacturing SMEs (Bellandi et al. 2019).

The continuing competitiveness of European manufacturing SMEs derives from their capacity to be innovative. However, because of their size and ownership structure, they also generally struggle with profitability and liquidity, thus becoming particularly vulnerable to external shocks (European Commission 2019). In fact, crises such as the COVID-19 pandemic are likely to have an inordinate effect on SMEs (Laufs and Schwens 2014; OECD 2009), given their limited resources (human, financial and technical) compared to large firms (Martin et al. 2019; Narula 2004). On the other hand, SMEs are more flexible and adaptable than their larger counterparts because of their small size, their tendency to be privately owned, and their relatively flat hierarchical structures, all of which can be beneficial during a crisis. Overall, however, SMEs are generally less resilient compared to larger firms, meaning that they take longer to return (if at all) to ‘normal operations’ following a crisis. This vulnerability became apparent in the aftermath of the 2008 global financial crisis, where SMEs experienced both a severe decline in demand and financial distress. The falling demand particularly affected manufacturing SMEs (OECD 2009; Wymenga et al. 2011). Hence, it is critically important to consider the role of manufacturing SMEs in recovering from the economic crisis associated with the COVID-19 pandemic.

This pandemic represents an external shock of unprecedented magnitude, affecting European SMEs on the supply and demand sides alike. For example, survey data from May 2020 suggest that 41% of UK SMEs had stopped operations and 35% feared they would be unable to reopen again (FSB 2020). In Germany, 50% of SMEs expected a negative effect due to the crisis with one-third anticipating a decline in revenues by more than 10% (DIHK 2020). In Italy, more than 70% indicated they were directly affected by the crisis (CNA 2020). While SMEs in other European countries have voiced similar concerns (OECD 2020), these firms are also highly heterogeneous along several dimensions. For example, in the context of the 2008 global financial crisis, Cowling et al. (2018) found that larger, established SMEs in the UK were more affected than smaller, younger SMEs. The latter were more agile and speedy in responding to the crisis, while older firms were not. Thus, while external shocks affect all SMEs to some extent, it is important to consider how different ‘types’ of SMEs are affected by a crisis.

In this paper, we examine how different types of European manufacturing SMEs are affected by the COVID-19 crisis and draw some policy implications based on the analysis. Clearly, the impact of this crisis will vary substantially across industries, and this will require detailed sectoral analyses and foresight studies. However, this is beyond the scope of this paper. Rather, we build on the taxonomy by Narula (2002), which distinguishes between three types of SMEs. Firstly, there are *stand-alone SMEs*, which trade under their own brand, producing final goods for the consumer or industrial markets. They can, for example, be found in the Italian and Spanish ceramic tile industry (Fernández-Mesa and Alegre 2015). More generally, stand-alone SMEs struggle to achieve economies of scale, and many of them seek to differentiate themselves, by operating in (global) niches. However, especially in traditional sectors where differentiation through product or process innovation is difficult to achieve, stand-alone SMEs are continuously challenged by directly competing with their larger counterparts.

Secondly, specialist-supplier SMEs primarily exist in oligopolistic environments where they provide intermediate goods to larger firms; for example, in the automotive and aerospace industries (Frigant 2013; Speldekamp et al. 2020). While some are entirely dedicated to one (or a few) customer(s), operating under exclusive customer–supplier agreements, others are part of GVCs, where there is a high degree of interdependence between actors.

Finally, knowledge-based SMEs generally develop emerging, pre-paradigmatic technologies, supplying specialized knowledge-based assets to other firms or consumer markets. Many operate in nascent sectors, such as biotechnology, medical research or ICT manufacturing, where they collaborate with (or spin-off from) universities and large multinationals.

## Implications of the COVID-19 pandemic for European manufacturing SMEs

Though the full economic impact of the pandemic is hard to predict, it is already apparent that it represents an unprecedented external shock. On the supply side, SMEs have faced logistical issues due to the disruption of transportation and labor shortages. On the demand side, SMEs have seen their demand decline substantially due to lockdown measures, a drop in consumer confidence and the shutting down of a number of GVCs in affected industries. The severity of these demand and supply shocks is likely to differ according to whether the firm is a stand-alone, a specialized-supplier or a knowledge-based SME. These nuances are important to better understand the medium- and long-term effects, and to set policies accordingly. It is worth distinguishing between the immediate effects of the shock, due to the almost-simultaneous universal lockdowns across major economies between April and June 2020, and its longer term effects.

### Immediate effects of the lockdowns on different types of manufacturing SMEs

SMEs of all types have experienced immediate effects of the lockdowns on their upstream and downstream activities. Specifically, on the supply side, stand-alone SMEs have faced considerable logistical issues. However, while reduced capacity utilization represents one key challenge, demand-side effects pose a more severe threat. Indeed, most consumer-focused, stand-alone SMEs operate in industries characterized by an elastic demand. With consumers facing employment uncertainties and financial constraints, many of these SMEs have experienced a sudden decline in demand. In contrast, many stand-alone SMEs serving the industrial market may benefit from their niche position, as they face few substitutes and, therefore, customers may be reliant on them. However, with production lines being halted across Europe during the lockdowns, and GVCs disrupted, many of these firms are also likely to face reduced demand or an incapacity to produce.

Specialist-supplier SMEs were also severely hit both on their demand-side and their supply-side. With many being engaged in exclusive agreements, orders from key customers which themselves stopped production were likely to be halted. Such effects have occurred in the automotive sector, for example. However, given their specialized focus, these SMEs supply vital components to their customers and, hence, once restrictions are lifted and production restarts, demand is likely to recover. Notably, some SMEs serving the industrial markets, including stand-alone and specialist-supplier SMEs, may have temporarily repurposed their operations towards goods needed during the pandemic, such as face masks and medical equipment; at least making up for some of their lost demand.

Finally, demand-side implications for knowledge-based SMEs during the crisis were likely to have been less severe, compared to their supply-side issues. With many of these SMEs requiring laboratories or special equipment, for example, little progress can be made with scientists and engineers having to work from home.

### Short- and long-term implications for different types of manufacturing SMEs

There are economic implications associated with the COVID-19 pandemic that go beyond the lockdowns. As outlined in Table 1, there are short-term effects which apply to all types of SMEs and there are medium- and long-term implications which differ across these firms. In the short-term, SMEs of all types will encounter financial concerns and liquidity issues. Specifically, stand-alone SMEs have experienced a rapid decline in demand as well as some supply chain challenges, making them particularly reliant on external financial support. Even before the crisis, many of them were operating under tight budgets and financial constraints. These have worsened as a result of the crisis, making their demise more likely. Knowledge-based SMEs and specialist-suppliers have also experienced some interruptions, making many of them vulnerable to financial issues as well. Additionally, in order to follow continued government restrictions associated with social distancing, many SMEs have had to implement significant changes in the physical establishments, requiring further financial investment. Thus, as shown in Table 1, there are important universal short-term effects associated with the pandemic.

**Table 1:** Short-term and longer term effects of the COVID-19 crisis on manufacturing SMEs

	Stand-alone SMEs (consumer and industrial)	Specialist-supplier SMEs	Knowledge-based SMEs
Short-term	Most SMEs face liquidity issues and tightened financial constraints, following the disrupted operations during the lockdowns There is an increased reliance on governmental subsidies and many SMEs apply for their local, governmental support schemes; depending on eligibility The main focus is on addressing immediate operational issues, ‘fixing’ particular disruptions on the demand and the supply side New concepts and changes are put in place to ensure further governmental restrictions are being followed; for example, ensuring social distancing measures		
Medium-/ Long-term	Many of these SMEs will have to upgrade their digital infrastructure, enabling online sale channels, teleworking etc Employees will have to be re-trained accordingly Long-term investments will be required to upgrade production processes to cut costs and increase productivity New opportunities will arise for industrial SMEs to enter new supply chains or deepen their involvement in existing ones; some may also re-organize their own supply chains	GVCs will be reorganized; growing trends towards multiple sourcing, stock management; reshoring, embracing geographical proximity Supplier SMEs will see changes on both sides: They will re-organize their own supply chains and will see their key customers reorganize Staying in existing agreements and entering new ones will depend on their ability to keep costs low and match prices; without compromising quality Long-term and increased investments in digital technologies will be required to enable automation	New applications of their technologies can arise in a post-COVID-19 world; for example, in areas such as medical research and equipment Some may adapt their nascent technologies towards finding solutions for and tackling the issues having arisen during and through the pandemic Successful SMEs can benefit from further governmental research grants New start-ups may emerge to capture areas of growth through ‘creative destruction’

In the longer term, however, other challenges (and opportunities) will arise that differ across types of manufacturing SMEs. In particular, the crisis has pointed stand-alone SMEs to the importance of investing in digital technologies. These became essential for SMEs, not only to support crucial downstream activities such as sales and marketing, but also to increase their internal efficiency and productivity (PwC 2018). From a managerial perspective, digitization provides new opportunities for firms (Cirillo and Zayas 2019) and the crisis associated with the COVID-19 pandemic will accelerate the responsiveness of SMEs to harness them. However, some SMEs may struggle to implement digital initiatives due to prevailing financial concerns following the crisis, and the need to re-train staff accordingly. Nevertheless, besides digitalization, the aftermath of the COVID-19 pandemic might also give a new impulse to other ongoing trends affecting manufacturing SMEs, such as the transition towards environmental sustainability.

Moreover, as outlined in Table 1, the COVID-19 pandemic is likely to change GVCs (UNCTAD 2020; Gereffi 2020). For decades, these have been characterized by a globally scattered production system, tailored towards cost-optimization through cheap labor and just-in-time production (Javorcik 2020). The pandemic has evidenced the need to make GVCs more ‘resilient’ through supply chain diversification and reversing the perceived overreliance on a few (Asian) suppliers, reducing the dangers of a monopsonistic situation in the future (Miroudot 2020). Changes in GVCs are likely to occur in terms of reshoring, supplier diversification, stock management and embracing proximity, which brings new opportunities for European specialist-supplier SMEs and industrial stand-alone SMEs (see Table 1). Specifically, the potential reshoring of certain aspects of manufacturing operations to Europe represents a key opportunity to be harnessed by these SMEs. However, participants in modern value chains are characterized by a high degree of specialization and customization. This level of specialization takes time, effort and money for new members to achieve, and requires coordination with many other actors. Thus, the high degree of interdependence between GVC actors makes the replacement of one member rather difficult (Shih 2020). Nevertheless, the COVID-19 pandemic is likely to provide an impulse for GVC reorganization.

It is likely, therefore, that some of the anticipated changes will require GVC ‘lead firms’ to reorganize their supply chains and some specialist-suppliers will see their contracts terminated. In general, SMEs in GVCs are particularly vulnerable to external shocks because ‘lead firms’ often pass their own difficulties on to them (OECD 2009; Narula 2019). Thus, to keep their position in GVCs, European specialist-supplier SMEs will have to be highly innovative to keep their costs down, whilst maintaining price and quality standards. Those SMEs with prior experience and investments in such cost-oriented process and organizational innovations enjoy a natural advantage, making their survival more likely. Additionally, a post-COVID-19 world will be characterized by greater digital customer interactions and automated processes, which may well enable flexible and stable supply chains (McKinsey 2020). Thus, in order to stay relevant in GVCs, European specialist-supplier SMEs will not only have to be highly innovative to match prices and quality standards of rivals, but also to upgrade their internal operations through digital initiatives.

Despite these challenges brought by changes in GVCs, there will also be entrepreneurial opportunities, offering new areas of growth and allowing newer SMEs to benefit from creative destruction dynamics (Schumpeter 1911). Indeed, empirical evidence suggests that highly ambitious entrepreneurs are able to perceive opportunities in times of adversity and exploit unfulfilled gaps and market needs following a crisis (Giotopoulos et al. 2017). For example, birth rates in ICT manufacturing, which prior to the pandemic were relatively low (European Commission 2017), may increase as a response to the anticipated demand for ICT equipment induced by teleworking. On the other hand, scholars argue that entrepreneurship is dependent on the economic climate and is likely to be particularly hit by external crises (Klapper and Love 2011). For example, empirical evidence points towards lower levels of entrepreneurship following the 2008 financial crisis (OECD 2009). With entrepreneurial endeavors ultimately being tied to uncertainty and financial risks, many potential entrepreneurs may put their plans on hold, until more concrete implications of the pandemic have unveiled. Overall, whether existing SMEs will survive and whether entrepreneurship can flourish will ultimately be determined largely by European policymakers.

## Policy responses: from survival to renewal and growth

European policymakers were quick to recognise that supporting SMEs was of paramount importance to address the economic crisis caused by the pandemic. In contrast to the 2008 financial crisis where initial policy attention in most European countries focused on supporting banks and large construction firms this time around SMEs have become the center of policy attention from the outset.

Recognizing that SMEs tend to be most vulnerable during this kind of severe economic shock, the immediate policy priority at the beginning of the pandemic has been to address the challenges of the *survival phase* for SMEs. This has  entailed different kinds of financial support to prevent liquidity crunches and minimize employment losses. Progressively, and as confinement measures are alleviated, policies will need to refocus towards the renewal and growth phase for those SMEs that survived, shifting to more structural policies aimed at promoting innovation, internationalization and networking. Table 2 summarizes the scope of these two phases of policy intervention and the main policy instruments used in each phase.

**Table 2:** Policy approaches to support manufacturing SMEs during the COVID-19 crisis

	Survival phase	Renewal and growth phase
Main focus	Financial support to prevent liquidity crises and maintain employment	Structural measures to foster innovation, internationalization and networking
Time horizon	Short-term	Medium-/Long- term
Target groups	One-size-fits-all approach	Differentiated strategy by sector and by type of SME
Policy mix	Reduction of working hours and temporary unemployment Deferral of tax, social security payments, debt payments, and rent and utility payments Loan guarantees Direct lending to SMEs Grants and subsidies	Support for internationalization Innovation support schemes Training and skills development Teleworking and digitalization Cluster development and networking initiatives Entrepreneurship and start-up support

A recent OECD survey offers a useful cross-country analysis of SME policy responses during the survival phase (OECD 2020). A remarkable finding is that most European governments acted quickly to protect the workers of SMEs through specific wage support schemes for partial unemployment, working hour reduction and sick leave. Most European countries also put in place measures that enabled SMEs to postpone payments, as well as new lines of finance to protect the liquidity of SMEs. The latter have included loan guarantees to incentivize commercial banks to expand their lending to SMEs, as well as direct lending to SMEs through new funds created by governments or central banks. In addition to loans, several European governments also offered direct financial support to SMEs through grants and subsidies. Altogether, the policy mix to support the survival of SMEs during the first stage of the crisis was the same, regardless of the type of SME. However, such policies were more critical to specialized-supplier SMEs, which suffered most from the disruption of GVCs, than to certain stand-alone SMEs with nationally-bound operations, particularly in certain sectors such as the agro-food industry or the manufacturing of medical equipment, which might have even benefited from the crisis.

In the renewal and growth phase, the focus of the policy mix needs to shift towards more structural measures aimed at promoting the resilience of SMEs in the longer term and supporting their further growth. The focus here is to foster innovation, internationalization and networking. In contrast with the survival phase, in this case different types of SMEs might require different policy interventions. Indeed, more strategic and targeted policy strategies will become necessary in this stage, taking into consideration the opportunities and challenges facing different sectors and the particularities of different types of SMEs within those sectors. For example, stand-alone SMEs will benefit more from policy support to enter new international markets, while the best policy approach towards specialized-supplier SMEs might be to strengthen local networks and clusters, promoting the embeddedness of their customers (often multinational firms) in the territory. All types of SMEs will benefit from strong innovation support schemes, but the focus required might be different. Specifically, product and marketing innovations are most relevant for stand-alone SMEs, including those that differentiate themselves (or operate in niches) and those which—due to the nature of their industry—do not. For the latter group, marketing innovations may be particularly valuable to retain existing and attract new customers. For specialized-suppliers, the main focus will need to be on process and organizational innovations, enabling them to compete on price and quality. Meanwhile, stronger investments in entrepreneurship and start-up support will turn critical to promote knowledge-based SMEs. The most adequate policy mix will also depend on the particularities of each industry and territory, making it necessary to adopt more strategic policy responses, in contrast with the one-size-fits-all approach that characterizes the survival stage.

The shift from a focus on survival to the renewal and growth phase is a delicate one that requires a proper sequencing of policy responses. While the survival phase is important, it is very costly and cannot last forever, given its profound effect on fiscal deficits and public debt. Removing the associated initial policy actions should, therefore, be well planned, gradually removing generic financial support measures, especially grants, as the economic situation stabilizes, while introducing new support policies fostering innovation and growth.

Policymakers will need to adopt a more proactive role, coordinating new industrial efforts for coupling domestic capacities with the dynamics of GVCs. As we have argued earlier, the COVID-19 crisis can also act as an accelerator of digitalization and sustainability transitions, while spurring a renewed interest in emerging health technologies such as biotechnology and genetics. Policymakers should create new incentives for European firms to benefit from these opportunities, against the context of the ongoing structural reshaping of globalization characterized by the increasing influence by China (Petricevic and Teece 2019). This calls for a new generation of demand-oriented innovation policies, such as strategic public procurement for innovation and investment in environmentally-oriented projects in the fields of renewable energy, transport infrastructure, and information technologies.

This approach -which has been increasingly influencing European, national and subnational policies over recent years- represents a shift towards “transformative” industrial and innovation policies, aimed at setting the direction of change rather than just addressing market failures (Schot and Steinmueller 2018). However, following the sharp fiscal effects of the initial wave of public expenditure in the “survival phase”, many European countries will lack the capacity to aggressively pursue this kind of strategic “renewal and growth” agendas. Therefore, policy intervention at the EU level will become critical to support European manufacturing SMEs, including new lines of research and innovation funding from the European Commission, as well as dedicated investments and financing by the European Investment Bank.

Given the contribution of manufacturing SMEs to the European economy, policymakers need to carefully reassess their policy mix in light of the evolution of the crisis. An ambitious structural policy approach based on promoting the renewal and growth of European manufacturing SMEs—through innovation, internationalization and networking—is of paramount importance to deal with the challenges ahead.

## Footnotes

1 The European Commission defines SMEs as firms with less than 250 employees and have an annual turnover of up to 50 million Euros, or a balance sheet of no more than 43 million Euros (as set out by the Commission Recommendation of 6 May 2003) (European Commission 2019).

## References

[CR1] Bellandi M, De Propris L, Santini E, Bianchi P, Durán CR, Labory S (2019). Industry 4.0 + challenges to local productive systems and place-based integrated industrial policies. Transforming industrial policy for the digital age: Production, territories and structural change.

[CR2] Cirillo V, Zayas JM (2019). Digitalizing industry? Labor, technology and work organization: an introduction to the Forum. Journal of Industrial and Business Economics.

[CR3] Cowling M, Liu W, Zhang N (2018). Did firm age, experience, and access to finance count? SME performance after the global financial crisis. Journal of Evolutionary Economics.

[CR4] CNA. (2020). Effetti negativi sul 72% delle imprese, oltre 7mila risposte al questionario CNA. https://www.cna.it/effetti-negativi-sul-72-delle-imprese-6-327-risposte-al-questionario-cna/. Accessed 26 May 2020.

[CR5] DIHK (2020). Auswirkungen des Corona-Virus auf die deutsche Wirtschaft: DIHK-Blitzumfrage März 2020, Deutsche Industrie- und Handelskammern.

[CR6] European Commission. (2017). *Annual report on European SMEs 2016/2017 Focus on self-employment*, Luxembourg.

[CR7] European Commission. (2019). *Annual report on European SMEs 2018/2019 Research and Development and Innovation by SMEs*, Luxembourg.

[CR8] Fernández-Mesa A, Alegre J (2015). Entrepreneurial orientation and export intensity: Examining the interplay of organizational learning and innovation. International Business Review.

[CR9] Frigant V (2013). Putting SMEs back at the heart of automotive supply chain analysis. International Journal of Automotive Technology and Management.

[CR10] FSB. (2020). One in three closed small firms fear they’ll never reopen amid widespread redundancy plans. National Federation of Self Employed and Small Businesses. https://www.fsb.org.uk/resources-page/one-in-three-closed-small-firms-fear-they-ll-never-reopen-amid-widespread-redundancy-plans.html

[CR11] Gereffi, G. (2020) What does the COVID-19 pandemic teach us about global value chains? The case of medical supplies. *Journal of International Business Policy*, **(forthcoming)**.

[CR12] Giotopoulos I, Kontolaimou A, Tsakanikas A (2017). Drivers of high-quality entrepreneurship: What changes did the crisis bring about?. Small Business Economics.

[CR13] Javorcik B, Baldwin RE, Evenett SJ (2020). Global supply chains will not be the same in the post-COVID-19 world. Covid-19 and Trade Policy: Why turning inward won't work.

[CR14] Kano L, Tsang EW, Yeung HWC (2020). Global value chains: A review of the multi-disciplinary literature. Journal of International Business Studies.

[CR15] Klapper L, Love I (2011). The impact of the financial crisis on new firm registration. Economics Letters.

[CR16] Laufs K, Schwens C (2014). Foreign market entry mode choice of small and medium-sized enterprises: A systematic review and future research agenda. International Business Review.

[CR17] Martin D, Romero I, Wegner D (2019). Individual, organizational, and institutional determinants of formal and informal inter-firm cooperation in SMEs. Journal of Small Business Management.

[CR18] McKinsey. (2020). Digital strategy in a time of crisis. https://www.mckinsey.com/business-functions/mckinsey-digital/our-insights/digital-strategy-in-a-time-of-crisis. Accessed 26 May 2020.

[CR19] Miroudot S, Baldwin RE, Evenett SJ (2020). Resilience versus robustness in global value chains: Some policy implications. Covid-19 and trade policy: Why turning inward won't work.

[CR20] Narula R, Contractor F, Lorange P (2002). R&D collaboration by SMEs: Some analytical issues and evidence. Cooperative strategies and alliances.

[CR21] Narula R (2004). R&D collaboration by SMEs: New opportunities and limitations in the face of globalisation. Technovation.

[CR22] Narula R (2019). Enforcing higher labor standards within developing country value chains: Consequences for MNEs and informal actors in a dual economy. Journal of International Business Studies.

[CR23] OECD (2009). The impact of the global crisis on SME and entrepreneurship financing and policy responses.

[CR24] OECD (2020). Tackling coronavirus (COVID-19): Contributing to a global effort. SME policy responses.

[CR25] Petricevic O, Teece DJ (2019). The structural reshaping of globalization: Implications for strategic sectors, profiting from innovation, and the multinational enterprise. Journal of International Business Studies.

[CR26] PwC. (2018). Europe Monitor Innovation and Digital Transformation: How do European SMEs perform? https://www.pwc.nl/nl/assets/documents/pwc-europe-monitor-innovation-sme.pdf. Accessed: 7 May 2020.

[CR27] Schot J, Steinmueller WE (2018). Three frames for innovation policy: R&D, systems of innovation and transformative change. Research Policy.

[CR28] Schumpeter J (1911). Theori der Wirtschaftlichen Entwicklung.

[CR29] Shih, W. C. (2020). Bringing Manufacturing Back to the U.S. Is Easier Said Than Done. *Harvard Business Review*. https://hbr.org/2020/04/bringing-manufacturing-back-to-the-us-is-easier-said-than-done. Accessed: 27 May 2020.

[CR30] Speldekamp D, Knoben J, Saka-Helmhout A (2020). Clusters and firm-level innovation: A configurational analysis of agglomeration, network and institutional advantages in European aerospace. Research Policy.

[CR31] UNCTAD (2020). Impact of the COVID-19 pandemic on Global FDI and GVCs: Updates analysis. Global investment trends monitor.

[CR32] van Tulder R, Verbeke A, Piscitello L, van Tulder R, Verbeke A, Piscitello L (2018). Introduction: International Business in the information and digital age an overview of themes and challenges. International Business in the information and digital age (Progress in International Business Research).

[CR33] Wymenga, P., Spanikova, V., Derbyshire, J., & Barker, A. (2011). *Are EU SMEs recovering from the crisis? Annual Report on EU Small and Medium sized Enterprises 2010/2011*, Rotterdam and Cambridge: European Commission.

